# Comparison of Different Histone Deacetylase Inhibitors in Attenuating Inflammatory Pain in Rats

**DOI:** 10.1155/2019/1648919

**Published:** 2019-01-27

**Authors:** Yu Mao, Jing Zhou, Xuesheng Liu, Erwei Gu, Zhi Zhang, Wenjuan Tao

**Affiliations:** ^1^Department of Anesthesiology, First Affiliated Hospital of Anhui Medical University, Jixi Road 218, Hefei, Anhui Province, China; ^2^School of Life Sciences, University of Science and Technology of China, Huangshan Road 443, Hefei, Anhui Province, China; ^3^Department of Head-neck and Breast Surgery, The First Affiliated Hospital of University of Science and Technology of China, Anhui Provincial Cancer Hospital, Hefei, Anhui Province 233004, China; ^4^School of Basic Medical Sciences, Anhui Medical University, Meishan Road 81, Hefei, Anhui Province, China

## Abstract

Histone deacetylase inhibitors (HDACIs), which interfere with the epigenetic process of histone acetylation, have shown analgesic effects in animal models of persistent pain. The HDAC family comprises 18 genes; however, the different effects of distinct classes of HDACIs on pain relief remain unclear. The aim of this study was to determine the efficacy of these HDACIs on attenuating thermal hyperalgesia in persistent inflammatory pain. Persistent inflammatory pain was induced by injecting Complete Freund's Adjuvant (CFA) into the left hind paw of rats. Then, HDACIs targeting class I (entinostat (MS-275)) and class IIa (sodium butyrate, valproic acid (VPA), and 4-phenylbutyric acid (4-PBA)), or class II (suberoylanilide hydoxamic acid (SAHA), trichostatin A (TSA), and dacinostat (LAQ824)) were administered intraperitoneally once daily for 3 or 4 days. We found that the injection of SAHA once a day for 3 days significantly attenuated CFA-induced thermal hyperalgesia from day 4 and lasted 7 days. In comparison with SAHA, suppression of hyperalgesia by 4-PBA peaked on day 2, whereas that by MS-275 occurred on days 5 and 6. Fatigue was a serious side effect seen with MS-275. These findings will be beneficial for optimizing the selection of specific HDACIs in medical fields such as pain medicine and neuropsychiatry.

## 1. Introduction

Chronic pain, a pathologic manifestation of many diseases [1–3], is the leading cause of years lived with disability worldwide [4, 5]. Although a large number of pharmacologic therapies have been approved, many patients with chronic pain are still inadequately treated. Of note, most chronic pain types, such as lower back pain and headache, have no identifiable medical explanation, making them more difficult to treat [1–3]. Recent animal models and clinical studies have indicated that epigenetic regulation plays an important role in the development or maintenance of persistent pain, thereby shedding light on a direction for the development of novel therapeutics for persistent pain by targeting epigenetic regulating systems [6, 7]. Importantly, some epigenetic agents have no analgesic tolerance after repeated administration [8].

Histone acetylation, regulated by the activity of histone acetyltransferases (HATs) and histone deacetylases (HDACs), is involved in the initiation of pain. To date, 18 HDAC genes have been identified and are divided into four phylogenetically derived classes [9, 10]. Class I HDACs consist of HDAC 1, 2, 3, and 8 isoforms, which are ubiquitously expressed and predominantly localized in the nucleus. Class II HDACs are divided into two subgroups, namely, class IIa (HDAC 4, 5, 7, and 9) and class IIb (HDAC 6 and 10); these enzymes are primarily cytosolic and can be shuttled between the cytoplasm and nucleus depending on the phosphorylation status. Class III HDACs comprise sirtuins, which are located in the nucleus, cytoplasm, and mitochondria. Class IV HDAC only contains one member, HDAC 11, which is localized in the nucleus [9]. The distribution of different types of HDACs may vary in different diseases including chronic pain. However, it is unclear whether HDACs have subtype specificity in the onset or maintenance of chronic pain. Therefore, the use of inhibitors for different types of HDACs may be useful for understanding the roles of different types of HDACs in chronic pain.

Animal and human studies have strongly implicated that histone deacetylase inhibitors (HDACIs) can modify the nociceptive response and have analgesic properties through the pharmacological modulation of acetylation [11–23]. In addition, the response to current pain-relieving compounds including opioid [24–26], nonsteroidal anti-inflammatory drugs [27, 28], tricyclic antidepressants [29, 30], and valproic acid (VPA) sodium [31] has been demonstrated to correlate with several epigenetic mechanisms [32]. Many HDACIs have been developed for research purposes, which have been approved for the treatment of malignant tumors [33] and inflammatory diseases [34, 35]. While the property of these compounds on analgesia is promising, the data of their safety and efficacy are limited. HDACIs have analgesic effects in various pain models by different routes of administration [11, 13, 15, 36]; however, the analgesic efficacy and side effects of different HDACIs are unknown.

Notably, most current HDACIs can produce side effects including fatigue, diarrhea, nausea, thrombocytopenia, and bone marrow toxicity [37–39]. Here, we focused on several HDACIs from different chemical classes to determine their effects on inflammatory hyperalgesia in rat models.

## 2. Materials and Methods

### 2.1. Animals and Pain Models

All animal procedures were conducted after protocol approval by the Biomedical Research Ethics Committee of University of Science and Technology of China. Wistar rats (males, 7–10 weeks old, weighing 200–300 g) were used in the studies. The rats were housed under standard conditions (12 h: 12 h day/night cycle, lights on between 8:00 am and 8:00 pm, *n*=5 − 6). Rats were randomized into groups before all behavioral studies according to computer-generated random numbers. A total of 5-6 animals were assigned to each experimental group. Rats were excluded if they were too weak to finish the behavioral test or died during the experiment. The intraplantar injection of 40 *µ*L CFA into the left hind paw was performed to induce persistent inflammatory pain [40]. The injected hind paw showed erythema and edema, which is an indicative of inflammation [40].

### 2.2. Drugs and Administration

Suberoylanilide hydoxamic acid (SAHA, 40 mg/kg in 1% DMSO; Cayman Chemical, Ann Arbor, Michigan, USA) [41, 42], trichostatin A (TSA, 1 mg/kg in 1% DMSO; Cayman Chemical, Ann Arbor, MI, USA) [43], dacinostat (LAQ824, 10 mg/kg in 1% DMSO; Cayman Chemical, Ann Arbor, MI, USA) [44], entinostat (MS-275, 40 mg/kg in 1% DMSO; Selleck Chemicals, Houston, TX, USA) [45], sodium butyrate (160 mg/kg in saline; Sigma-Aldrich, St. Louis, MO, USA) [46], 4-phenylbutyric acid (4-PBA, 500 mg/kg in 1% DMSO; Sigma-Aldrich) [47, 48], and VPA (200 mg/kg in saline; Cayman Chemical, Ann Arbor, MI, USA) [49] were administered intraperitoneally 1 hour after CFA injection and once daily for 3 days, and control rats received saline or 1% DMSO as vehicle injection. Animals received HDACIs or vehicle (1% DMSO) via intraperitoneal injection daily for 3 days. An experimenter blinded to the assignment and injection performed the subsequent testing.

### 2.3. Behavioral Analysis

**Figure 1:**
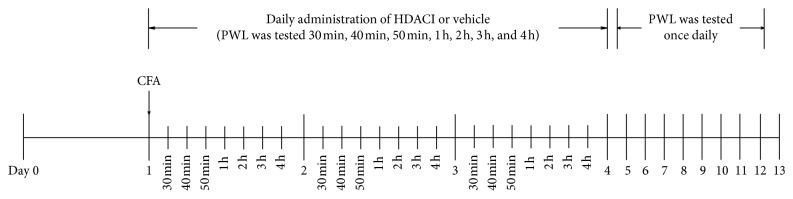
Flow chart of the study evaluating the effects of HDACIs on thermal hyperalgesia in rats.

Before testing, animals were habituated to the Hargreaves apparatus (IITC Life Science Inc., Woodland Hills, CA, USA) [50] and the experimenter for at least 2 days. The baseline of paw withdrawal latency (PWL) to noxious heat stimulation was tested 1 day before HDAC injection. After 30 min of acclimation on a temperature-controlled glass platform in a clear plastic enclosure, a radiant light was directed towards the plantar surface of the left hind paw. A 20 s cutoff was applied to prevent tissue damage. Three measurements were done for each animal per test session separated by 90 s. The PWL value for baseline was tested the day before CFA injection. After creating the inflammatory lesion, PWL was tested 30, 40, and 50 min, and 1, 2, 3, and 4 h after injection of HDACIs or vehicle for at least 3 days. One additional day for HDACI injection and testing was conducted for some compounds without significant attenuation of thermal hyperalgesia between HDACI and vehicle during the first 3 days. Then, a once daily PWL test was accomplished until inhibition of hyperalgesia was abrogated (Figure 1). The mean value of PWL at several time points each day during the first 3 or 4 days after CFA injection was used to assess the daily change in thermal sensitivity thresholds.

### 2.4. Statistical Analysis

All data are presented as the mean ± standard error of the mean (SEM). Two-way analysis of variance (ANOVA) followed by the Bonferroni posttest was used for statistical analysis of behavioral data between two groups at the same time point. One-way ANOVA with post hoc Tukey's test was applied for statistical analysis of the data between SAHA and other tested HDACIs. *P* < 0.05 was considered statistically significant.

## 3. Results

**Figure 2:**
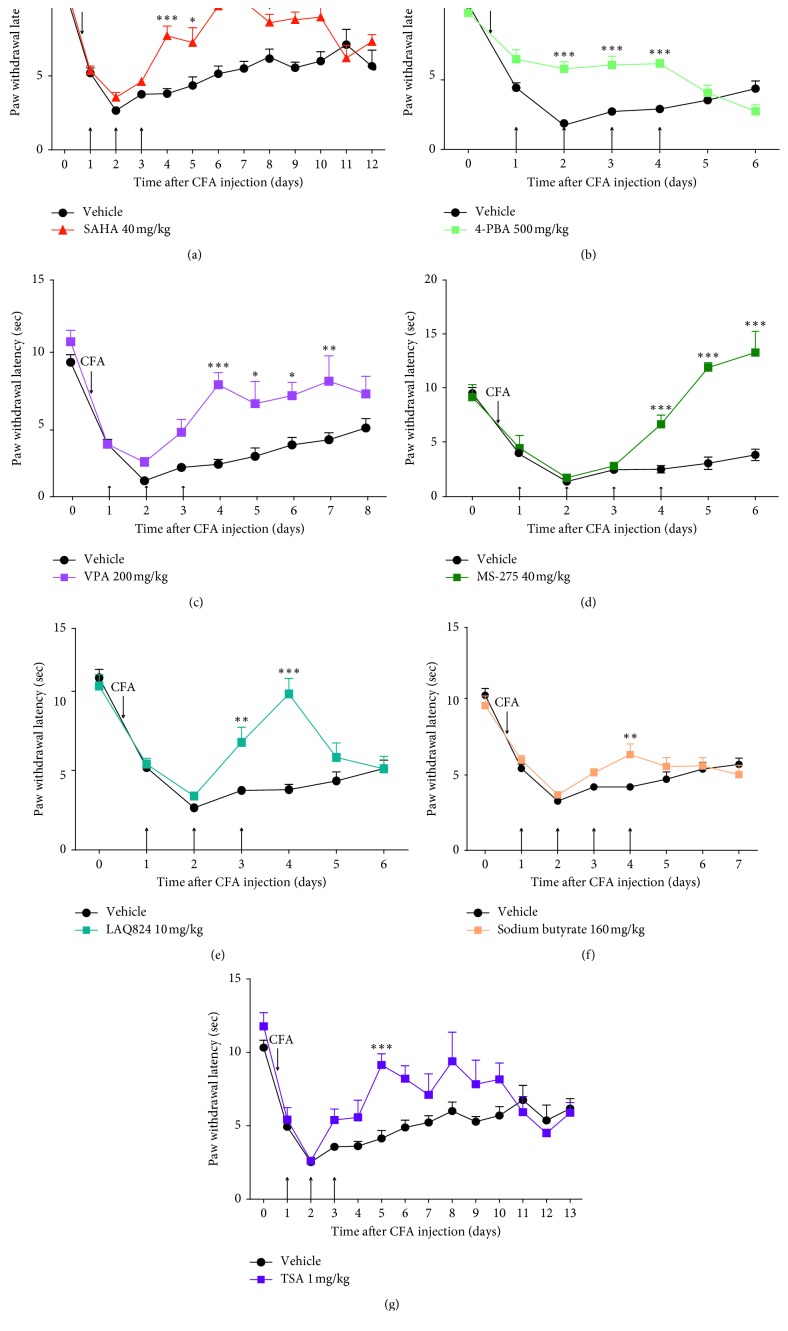
Attenuation of effects of postinjected HDACIs on thermal hyperalgesia in rats. Rat received injection of HDACI or vehicle (arrows show the time of injection) at the indicated dose after unilateral intraplantar injection of CFA. PWL was determined before CFA injection as a baseline and after CFA injection as hyperalgesia response. SAHA (a), TSA (b), LAQ824 (c), sodium butyrate (d), 4-PBA (e), VPA (f), and MS-275 (g) were injected for the panel (in parenthesis). Inhibition of hyperalgesia by all HDACIs was calculated by two-way ANOVA. Data show PWL as the mean ± SEM for the ipsilateral paw (^*∗*^*p* < 0.05, ^*∗∗*^*p* < 0.01, and ^*∗∗∗*^*p* < 0.001 compared with vehicle-treated rats at the same time point).

Suberoylanilide hydoxamic acid (SAHA), which has been approved for clinical use in lymphoma, is believed to target class I, II, and IV HDACs [6, 51, 52] and was shown to reduce hyperalgesia in an animal model of inflammatory pain after intrathecal injection drug administration [11, 13, 15]. We first tested SAHA in Complete Freund's Adjuvant (CFA)-induced persistent inflammatory pain in rats. After inflammatory lesions were created on day 1, paw withdrawal latency (PWL) was tested 30, 40, and 50 min, and 1, 2, 3, and 4 h after the injection of SAHA for the first 3 days and once daily for the next 9 days (Figure 1). As shown in Figure 2(a), after 3 days of SAHA administration, thermal hyperalgesia was significantly alleviated from day 4 and lasted 7 days.

Next, we tested other groups of inhibitors specific to different classes of HDACIs. Trichostatin A (TSA) and dacinostat (LAQ824) target class I, II, and IV HDACIs; sodium butyrate, 4-phenylbutyric acid (4-PBA), and VPA inhibit class I and IIa HDACIs; and entinostat (MS-275) suppresses class I HDACIs [6, 11, 53–56]. Suppression of hyperalgesia by 4-PBA had rapid onset from day 2 after administration and disappeared on the day when treatment stopped, so repeat injection of 4-PBA was performed for 4 days (Figure 2(b)). In contrast, VPA and MS-275 attenuated hyperalgesia from day 4 and lasted 4 and 3 days, respectively (Figures 2(c) and 2(d)). Attenuation of hyperalgesia by LAQ824 was sustained for 2 days from day 3 after HDACI injection (Figure 2(e)). Sodium butyrate and TSA reduced CFA-induced thermal hyperalgesia on days 4 and 5 after HDACI injection, respectively (Figures 2(f) and 2(g)). Sodium butyrate and MS-275 were both injected for 4 days because no significant attenuation of thermal hyperalgesia was observed during the first 3 days (Figures 2(d) and 2(f)).

**Figure 3:**
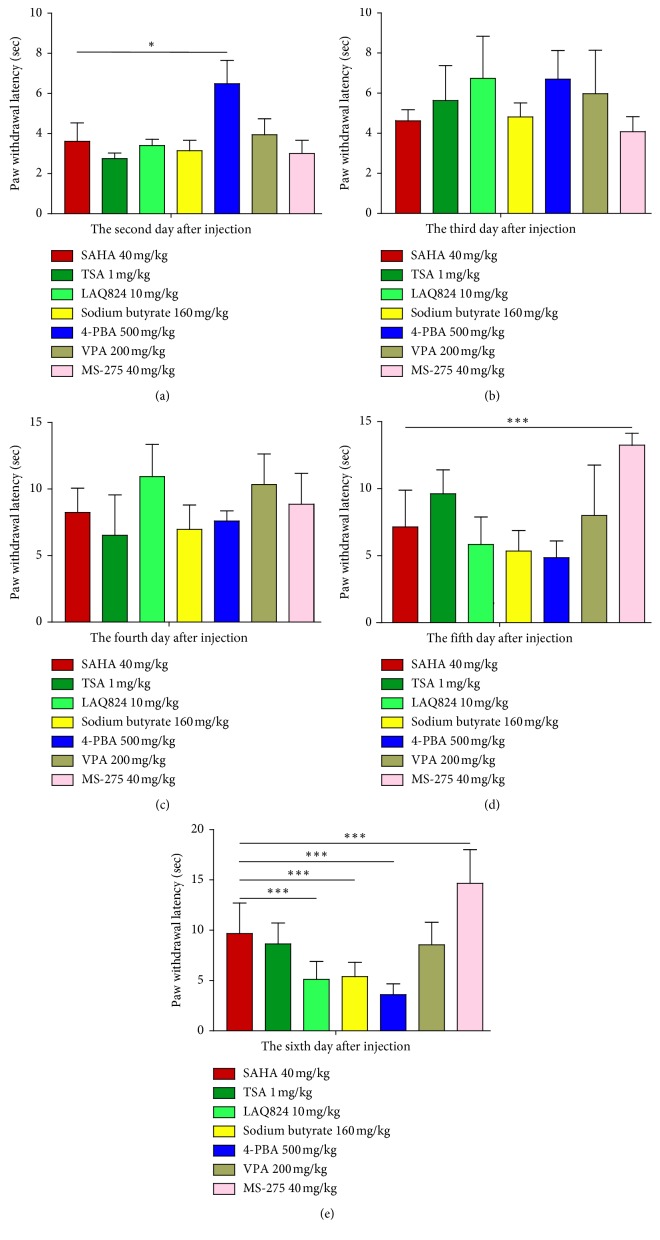
Comparison of SAHA with other tested HDACIs in inhibition of thermal hyperalgesia after intraplantar injection of CFA. Inhibition of hyperalgesia by all HDACIs displayed from the second to sixth day after CFA injection was calculated using one-way ANOVA. Data show PWL as the mean ± SEM for the ipsilateral paw (*∗p* < 0.05, and *∗∗∗p* < 0.001 compared with SAHA-treated rats at the same time point).

As shown in Figure 2, SAHA led to the longest inhibition of thermal hyperalgesia among the tested HDACIs. We questioned whether it was the strongest HDACI for diminishing the withdrawal response from days 2 to 6 after CFA injection. In comparison with SAHA, 4-PBA more notably suppressed hyperalgesia on day 2 (Figure 3(a)); however, this effect gradually declined on day 6 (Figure 3(e)). It is of great interest to note that compared to SAHA, the extent of MS-275 in reducing hyperalgesia remained obvious on days 5 and 6 (Figures 3(d) and 3(e)). Other agents including TSA, LAQ824, sodium butyrate, and VPA did not show a significant advantage over SAHA in inhibiting hyperalgesia after CFA injection (Figure 3).

## 4. Discussion

In this study, we characterized the effects of different types of HDACIs on analgesia in rat models of inflammatory pain. Among them, the analgesic effects of 4-PBA had a fast onset and short duration of action, while SAHA had the longest effect in suppressing hyperalgesia that lasted for up to 7 days. Severe fatigue was the most common side effect of MS-275 [57, 58].

Paw edema and hyperalgesia are indeed signs of inflammation. However, they are not always go hand in hand. It has been shown that inflammatory hyperalgesia can be reversed without ameliorating the edema [59]. Certainly, mechanical hypersensitivity was also affected by some of the HDACI used in this study. We tested thermal pain in the current study because most of HDACI studies on pain sensitization were performed by this manner in previous studies [11, 60, 61]. Because the aim of this study was to test the effects of different HDACIs on pain, we need to compare our results with those in previous studies, which could be an excellent control. Different HDACI administration routes result in a high degree of variability in the results in animal models, and no comparison could be further analyzed between different pain types [11–13, 15, 60, 62]. Considering the possibility of comparing and translating the data to humans, the route of drug administration in this study was intraperitoneal administration. Furthermore, all tested compounds retained the ability to cross the blood-brain barrier (BBB) [63, 64]. In this study, we found that SAHA produced long-term attenuation of CFA-induced thermal hyperalgesia, while the short-term reduction was induced by TSA, LAQ824, sodium butyrate, 4-PBA, VPA, and MS-275. Although Table 1 lists the sensitivities of tested drugs to the isoforms of HDAC classes [63, 65], our results provided different HDACI efficacies in rat models of persistent inflammatory pain. These results may provide a reference for the potential role of a specific HDAC subtype in the pathogenesis or maintenance of chronic pain in a predictive model. In addition, since there are great differences in the mechanisms of different types of chronic pain, further research is needed on whether the analgesia profile of different HDACIs is the same in persistent inflammatory pain.

Notably, SAHA was only effective after a 3-day treatment, which may reflect the SAHA-sensitive HDAC modification in the development of peripheral or central sensitization by persistent inflammation. Zhang et al. [13] reported that SAHA repeatedly injected (once a day for 4 days) into the nucleus raphe magnus relieved CFA-induced thermal hyperalgesia from days 4 to 8 after injection through enhanced expression of glutamic acid decarboxylase 65. However, Bai et al. [11] reported that intrathecal injection of SAHA corresponding with enhanced histone acetylation in the spinal cord. These studies suggest that there may be alternative mechanisms by which SAHA modulates pain relief. HDACIs not only regulate acetylation of histone but also modify the acetylation of other genes involved in nociceptive processing [15, 18, 36, 66–68]. One representative example demonstrates the importance of metabotropic glutamate receptor 2 (mGluR2) correlating with acetylation of p65/RelA [15]. Chronic (but not acute) treatment of SAHA reduced inflammatory pain, which corresponded with the upregulation of mGlu2 in the spinal cord and the dorsal root ganglion. SAHA also inhibits inflammatory processes by reducing Toll-like receptor-mediated activation of nuclear factor-κB p65 [10, 69]. Thus, SAHA targeting both histone and nonhistone acetylation events may be a promising drug for the treatment of inflammatory pain.

Besides acting on HDACs, VPA may act through several different mechanisms: enhanced GABAergic signaling [70, 71], increased serotonergic inhibition and reduced NMDA-receptor-mediated glutamate excitation [72], and inhibition of voltage-dependent sodium channels [73]. Such multivalent actions may improve VPA on attenuating CFA-induced thermal hyperalgesia with reduced side effects. Subcutaneous injection of MS-275 reduced the nociceptive response in animal models of inflammatory pain, which was consistent to our study. In contrast, Bal et al. [11] stated that intrathecal injection of MS-275 failed to attenuate thermal hyperalgesia. This difference was mainly due to the route of administration. The advantage of intrathecal injection is that it allows most drugs to bypass the BBB, resulting in HDAC inhibiting only in the spinal cord and the brain. By contrast, the obvious advantage of systemic administration is that it allows all tissues along the nociceptive pathway from the periphery to the brain. Preemptive injection of MS-275 prior to trigeminal inflammatory compression not only reduced the duration and magnitude of whisker pad mechanical hypersensitivity but also prevented the development of persistent pain by modifying the gene overexpressing involved in peripheral nerve regeneration [74]. Therefore, systems other than the central nervous system may be targeted by MS-275 [75]. Although the ability of MS-275 to attenuate thermal hyperalgesia may be superior to SAHA on days 5 and 6 after CFA injection, it cannot be ignored that MS-275 led to serious side effects, which was mostly due to its toxicity at a high dose [57]. Novel and more potent HDACIs with high specificity for certain HDAC informs on specific tissues or cells may have better therapeutic outcomes and fewer adverse effects.

## 5. Conclusions

In summary, our data provide an analgesic profile of distinct HDACIs in a rat model of inflammatory pain, although their mechanisms remain unknown. These results provide important evidence for researchers to choose different HDACIs as a tool in future works.

## Figures and Tables

**Table 1:** Sensitivities of tested HDACIs to the isoforms of classes of HDACs.

HDAC	Class I				Class IIa				Class IIb	
	1	2	3	8	4	5	7	9	6	10
SAHA	**+**	**+**	**+**	**+**	**+**	**+**	**+**	**+**	**+**	**+**
TSA	**+**	**+**	**+**	**+**	**+**	**+**	**+**	**+**	**+**	**+**
LAQ824	**+**	**+**	**+**	**+**	**+**	**+**	**+**	**+**	**+**	**+**
SB	**+**	**+**	**+**	**+**	**+**	**+**	**+**	**+**		
4-PBA	**+**	**+**	**+**	**+**	**+**	**+**	**+**	**+**	**+**	**+**
VPA	**+**	**+**	**+**	**+**	**+**	**+**	**+**	**+**		
MS-275	**+**									

## Data Availability

The datasets generated and analyzed to support the findings of this study are available from the corresponding author on reasonable request.
