# Corrigendum to “Comparison of Different Histone Deacetylase Inhibitors in Attenuating Inflammatory Pain in Rats”

**DOI:** 10.1155/2021/9760961

**Published:** 2021-05-24

**Authors:** Jing Zhou, Yu Mao, Xuesheng Liu, Erwei Gu, Zhi Zhang, Wenjuan Tao

**Affiliations:** ^1^Department of Head-neck and Breast Surgery, Western district of the First Affiliated Hospital, University of Science and Technology of China, Hefei City, Anhui 233004, China; ^2^Department of Anesthesiology, First Affiliated Hospital of Anhui Medical University, Jixi Road 218, Hefei City, Anhui, China; ^3^School of Life Sciences, University of Science and Technology of China, Huangshan Road 443, Hefei City, Anhui, China; ^4^School of Basic Medical Sciences, Anhui Medical University, Meishan Road 81, Hefei City, Anhui, China

In the article titled “Comparison of Different Histone Deacetylase Inhibitors in Attenuating Inflammatory Pain in Rats” [1], the order of the authors' list was incorrect in the original publication and the correct order of the authors' list is shown above.
